# An Overview of Potential Seaweed-Derived Bioactive Compounds for Pharmaceutical Applications

**DOI:** 10.3390/md20020141

**Published:** 2022-02-15

**Authors:** Silvia Lomartire, Ana M. M. Gonçalves

**Affiliations:** 1 University of Coimbra, MARE—Marine and Environmental Sciences Centre, Department of Life Sciences, Calçada Martim de Freitas, 3000-456 Coimbra, Portugal; silvia.lomartire@student.uc.pt; 2 Department of Biology, CESAM—Centre for Environmental and Marine Studies, University of Aveiro, 3810-193 Aveiro, Portugal

**Keywords:** seaweed, bioactive compounds, pharmaceutical application, sustainability, health benefits

## Abstract

Nowadays, seaweeds are widely involved in biotechnological applications. Due to the variety of bioactive compounds in their composition, species of phylum Ochrophyta, class Phaeophyceae, phylum Rhodophyta and Chlorophyta are valuable for the food, cosmetic, pharmaceutical and nutraceutical industries. Seaweeds have been consumed as whole food since ancient times and used to treat several diseases, even though the mechanisms of action were unknown. During the last decades, research has demonstrated that those unique compounds express beneficial properties for human health. Each compound has peculiar properties (e.g., antioxidant, antimicrobial, antiviral activities, etc.) that can be exploited to enhance human health. Seaweed’s extracted polysaccharides are already involved in the pharmaceutical industry, with the aim of replacing synthetic compounds with components of natural origin. This review aims at a better understanding of the recent uses of algae in drug development, with the scope of replacing synthetic compounds and the multiple biotechnological applications that make up seaweed’s potential in industrial companies. Further research is needed to better understand the mechanisms of action of seaweed’s compounds and to embrace the use of seaweeds in pharmaceutical companies and other applications, with the final scope being to produce sustainable and healthier products.

## 1. Introduction

Our planet is covered by 70% aquatic habitats, inhabited by a great variety of organisms. Most of them are incredibly important and provide us with ecosystem services such as seafood, raw materials, tourism and cultural heritage. Macroalgae, or seaweeds, provide us with several services; they have long since been used as a nutritional food resource and in traditional medicine [1], but only in the last decades, with advanced technologies, has it been possible to characterize and apply the biological properties of macroalgal compounds for biotechnological purposes. Seaweeds are a source of peculiar compounds with interesting properties that can be useful for pharmaceutical and industrial applications. Due to their non-toxic, edible, cheap and easy culturing properties, macroalgae are optimal candidates for replacing synthetic compounds with those of natural origin.

The trend of eating seaweed-based food has increased as scientific reports have confirmed the antioxidant, antimicrobial and antiviral effects of seaweed metabolites. The potentiality of seaweed can vary depending on the type of algae, harvesting period and environmental conditions; thus, every species has peculiar compounds that can act in different ways, exhibiting diverse properties.

Through the present review, the main seaweed compounds will be discussed, and several study cases will be analysed to prove the beneficial effects of seaweed compounds for human health [2,3]. The most important polysaccharides found in brown seaweeds are fucoidan and alginate, along with carrageenan and agar from red seaweeds and ulvan in green seaweeds [2]. Some sulphate polysaccharides have an excellent hydrocolloidal property, by creating viscous liquid. Indeed, agar and carrageenan are widely involved in the commercial food process industry due to their ability to act as stabilizers, emulsifiers and thickening agents. They are already used in gel-based food products such as desserts, jams, jellies and bakery products [3].

Seaweeds are a great source of protein, minerals, vitamins, dietary fibre, antioxidants and essential fatty acids possessing low caloric value [4,5]; indeed, they have been easily incorporated in the development and formulation of nutraceutical food products. In addition, studies have proved that the inclusion of seaweeds in daily alimentation is associated with low incidence of numerous diseases and provides benefits to digestive health and chronic diseases such as diabetes, cancer and cardiovascular diseases [6,7,8], as well as bacterial and viral infections [9,10].

Therefore, the addition of seaweed compounds for the formulation of novel natural drugs is one of the aims of marine pharmaceuticals, a new branch of pharmacology, which has developed over the last decades.

The information gathered to produce the present review have been searched using the search engine Google Scholar, by using keywords such as “seaweeds and pharmacology”, “bioactivity of seaweeds compounds” and “seaweeds in traditional medicine”. More accurate research has been conducted using as key words seaweed’s bioactive compounds involved in pharmaceutical trials (fucoxanthin, fucoidan, carrageenan, agar, phenols, ulvan, caulerpin), with a particular attention to pre-clinical trials performed during the last decade.

## 2. Socio-Ecological Relevance of Seaweeds and Classification

Marine algae or seaweeds are multicellular photosynthetic primary producers widely distributed in the aquatic food chain. They are considered a fundamental component of the ecosystem as they are responsible for providing oxygen, food resources and shelter substrates for various organisms. Moreover, they provide in lowering the ocean acidity, being a possible solution to global warming [11,12,13,14]. 

In Asian regions as China, Japan or Korea algae have been consumed as a whole food or ingredient since ancient times for their nutritional benefits [15]. From a nutritional point of view, they are characterized by a high content of carbohydrates (<60%) and proteins (17–44%), a low percentage of lipids (<4.5%) and high presence of other micronutrients, such as vitamins, pigments and minerals.

Several seaweeds are potential candidates for biotechnological applications due to their characteristics. Their adaptation to extreme conditions increases their mechanisms of defence, thus, compounds responsible for the maintenance of seaweeds in harsh conditions might add value to the development of pharmaceutical bioproducts in order to fight diseases [16]. In the last decades, substances exclusive to seaweeds have been extracted in Europe, with the scope to substitute synthetic compounds with natural equivalent in the food, cosmetics industries and pharmaceuticals [17,18,19]. However, the great abundance of essential minerals and biochemical content in seaweeds may vary depending on the taxonomic group, geographical, seasonal and physiological variations [20,21,22]. Seaweeds are classified based on type and morphology, thus different genera and species have variation in bioactive compound types and functions [23]. They are mainly divided into three categories: Rhodophyta (red algae), Chlorophyta (green algae) and Ochrophyta (class Phaeophyceae, brown algae) [24,25].

Brown algae belong to a very large group of marine algae, with pigmentation that varies from yellow to dark brown [26]. Red algae contain high amounts of pigments such as carotenoids, chlorophyll (*a* and *d*), phycoerythrin, phycocyanin and allophycocyanin [27,28]. Green algae are mainly characterized with chlorophyll, a greenish lipid-soluble pigment commonly found in plants, algae and cyanobacteria. Chlorophyll plays a key role in photosynthesis and several biological functions [29].

The main pigments of seaweeds are classified into three groups: chlorophyll, carotenoid and phycobiliproteins. Carotenoids are widely distributed in nature. They include more than 1100 molecules [30], divided into two classes: xanthophylls, which contain oxygen, and carotenes, which are pure hydrocarbons. In photosynthetic organisms such as plants and algae, carotenoids absorb energy for photosynthesis and protect chlorophyll from photodamage [31]. The main carotenoids present in algae are carotenes, lycopene, fucoxanthin, astaxanthin, zeaxanthin, lutein, neoxanthin and violaxanthin. Fucoxanthin is one of the most abundant carotenoid found in edible brown algae and contributes over 10% total production of carotenoids in nature [29]. 

Phycobiliproteins are a group of water-soluble pigments, distinguishing three classes of molecules with different protein structure: phycocyanins (blue pigment), allophycocyanins (light blue pigment) and phycoerythrins (red pigment), this latter being the most abundant. Most pigments are already used as natural food colourant. Pigments not only play an important role in photosynthesis, but they also prevent plants and algae from UV radiations and cell damages and play an important role in several biological activities, thus it might be possible to involve pigments in pharmaceutical applications [29,32].

### Bioactive Compounds of Seaweeds and Their Potential to Ameliorate Human Health and Welfare

Functional compounds extracted from marine macroalgae possess several biological activities with beneficial properties [33,34] (Table 1). Extracts from seaweeds are considered to develop functional food and beverages [35,36,37], indeed analysis on green, red and brown algae revealed the presence of minerals, vitamins A, C, D, E, B_12_ and several compounds with interesting properties [38,39]. Marine algae are a primary source of iodine and bromine; indeed, the uptake of seaweeds can provide the daily iodine requirement of 150 mg/day [6]. All seaweeds can concentrate iodine from seawater, although the iodine content depends on species, stage of growth, season and geographical location. Generally, the iodine content of brown seaweeds is higher than red or green seaweeds, such as kelps (*Laminaria* sp., *Ascophyllum* sp., *Fucus* sp.) [40,41].

Romarís-Hortas et al. [42] report the concentration of iodine and bromine found in several seaweeds, namely *Himanthalia elongata*, *Saccorhiza polyschides*, *Laminaria ochroleuca* and *Undaria pinnafitida* as brown seaweeds, *Palmaria palmata* and *Porphyra umbilicalis* as red seaweeds and *Ulva rigida* as green seaweed. Results showed that iodine concentration was between 27.3 and 6138 µg/g, while bromine concentration was between 12.4 and 972.1 µg/g, with *Laminaria ochroleuca* being the species with the highest concentration of iodine and bromine. *Porphyra umbilicales* and *Porphyra linearis* shows similar iodine and bromine contents [42,43].

The occasional consumption of seaweeds as a source of iodine is recommended to treat iodine deficiency in the organism. Nevertheless, the regular absorption of iodine might give adverse effects on thyroid function, particularly in those with pre-existing thyroid disorder, pregnant women and neonates [44]. Concerning bromine, this element is not currently considered to be essential for health, although certain therapeutic uses of bromine as an anti-epileptic agent have been reported in some paediatric cases [45]. Moreover, the widespread production and use of brominated flame retardants has increased the interest on bromine compounds, which can be extracted from seaweeds [46]. Macroalgae are also a great source of polyunsaturated fatty acids (PUFAs) that consist in long hydrocarbon chains which terminate with hydroxyl groups. PUFAs are classified depending on the position of the first carbon–carbon double bond in omega-3 or omega-6. The main omega-3 FAs present in seaweeds are α-linolenic acid (ALA, 18:3n−3), eicosapentaenoic acid (EPA, 20:5n−3) and docosahexaenoic acid (DHA; 22:6n−3) [47]. 

EPA and DHA are considered the two most important PUFAs of marine lipids and they earn particular interest in animal diets due to the added nutritional value, antimicrobial and antioxidant properties. Moreover, EPA and DHA are gaining more attention due to their ability to give beneficial effects in conditions of cardiovascular disorders, Alzheimer’s disease, hypertension, coronary artery disease, arthritis and cancer [48,49,50]. 

The study of Gosch et al. [51] shows the variation of fatty acids profiles among brown, red and green seaweeds, where green seaweeds contain high levels of omega-3 FAs, in particular a high amount of ALA (data confirmed by Rohani-Ghadikolaei et al. [52]). The study reports the biochemical composition of seaweeds from the Persian Gulf. The authors suggest green seaweeds as potential food or feed additive for human and animal consumption. Moreover, biochemical analyses performed on Arabian Gulf Seaweeds identify *Ulva* spp. as the most suitable candidates for developing low-fat foods with PUFA-rich nutraceuticals [53].

Nevertheless, analysis from tropical seaweeds from the Indian coast reveal Phaeophyceae and Rhodophyta as being rich in omega-3 PUFAs and omega-6 PUFAs, respectively, while Chlorophyta are rich in monounsaturated fatty acids (MUFAs) [54].

Khan et al. [55] isolated two anti-inflammatory omega-3 PUFAs of stearidonic acid (SA) and EPA and one pro-inflammatory omega-6 PUFA of arachidonic acid (AA) from *Undaria pinnatifida* (Phaeophyceae). Results showed that SA and EPA reduced edema, erythema and blood flow potently in induced mouse ear inflammation, suggesting *Undaria pinnatifida* as remedy for inflammation-related symptoms. Therefore, future studies are needed to understand the environmental and seasonal impacts on FA profiles on seaweeds to obtain the right yield for biotechnological applications.

Marine algae are the most significant sources of non-animal polysaccharides, and their structures vary according with the type of algae, phase of life cycle of macroalgae when harvested and environmental conditions [56,57]. Polysaccharides (PSs) are carbohydrate biopolymers consisting of simple sugars linked by glycoside. The functional activities of PSs have been widely described in the literature as they show powerful biological activities. For example, isolate PSs (fucoidans) from Mediterranean brown seaweeds showed anti-inflammatory and gastroprotective activities [58]. Porphyran, a sulphated carbohydrate derived from red algae of the genus *Porphyra* showed anti-inflammatory, antioxidant, antihyperlipidemic, antioxidant and antitumour activities [59,60,61]. Immunostimulatory activity has been confirmed also in ulvan, a polysaccharide present in green algae [62]. Significant polysaccharides found in marine algae are agar, carrageenan, alginate, fucoidan, laminarin and ulvan [47].

**Figure 1:**
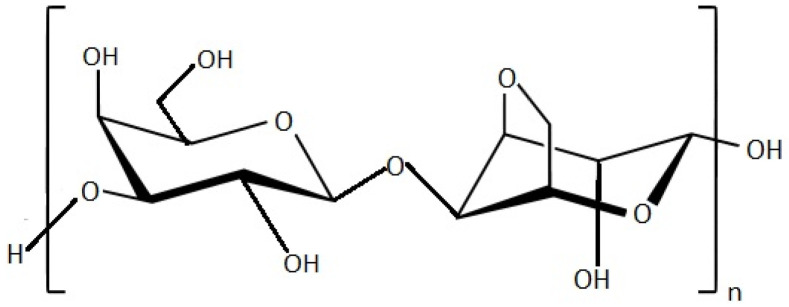
Chemical structure of agarose polymer.

Agar is a strongly gelling seaweed PS with hydrocolloidal properties. The main structure of agar is chemically characterized by the repeating units of D-galactose and 3,6-anhydro-L-galactose, with a few variations, as well as by a low ester sulphate content [63,64]. Matsuhashi [65] showed the structure of agar which consists in two groups of PSs: agarose (Figure 1), a neutral PS, and agaropectin, an oversimplified term for the charged PS.

**Figure 2:**
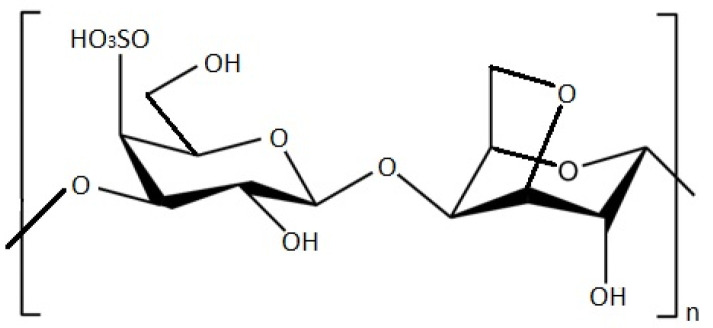
Chemical structure of κ-carrageenan.

Carrageenan, mainly found in red algae, is a linear PS formed from alternating sulphated or non-sulphated galactose units and linked with α-1,3-glycosidic bond and β-1,4-galactose bonds (Figure 2) [66]. Carrageenans are found in nature in the form of hybrids, as a mix of nonhomologous polysaccharides. Therefore, based on the principal repeated disaccharide unit and the position at which the sulphate group is connected on the galactose unit, carrageenans are divided into three major fractions (κ-carrageenan, ι-carrageenan and λ-carrageenan) and four minor fractions (θ-carrageenan, ν-carrageenan, ξ-carrageenan and µ-carrageenan) [67,68].

**Figure 3:**
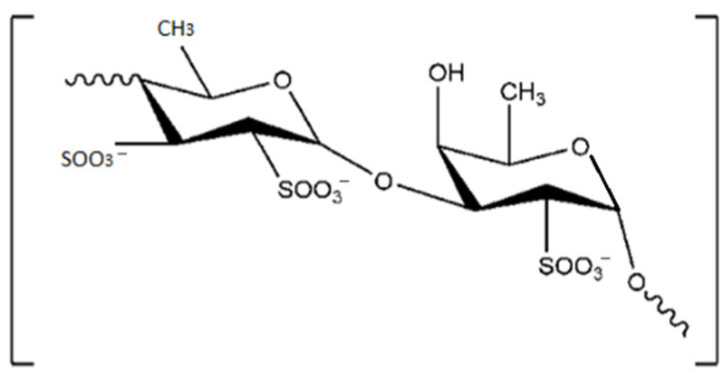
Chemical structure of PS fucoidan.

Fucoidan, alginate and laminarin are PSs found only in brown seaweeds. Fucoidans contain substantial percentages of L-fucose and sulphate ester groups (Figure 3). Their complex structures differ from species to species. Besides fucose and sulphate, they also contain other monosaccharides (mannose, galactose, glucose, xylose, etc.) and uronic acids, even acetyl groups and protein [69].

**Figure 4:**
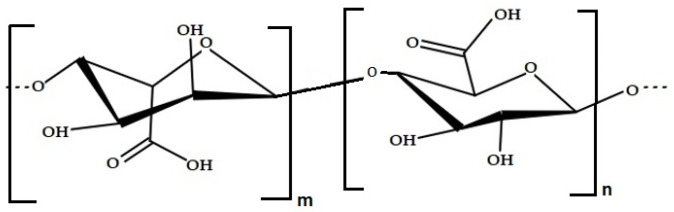
Chemical structure of alginic acid.

Alginate is a polysaccharide made of polymers of alginic acid, with monomer units of β-D-mannuronic acid (M) and α-L-guluronic acid (G) joined by 1,4 linkages [70,71,72] (Figure 4). Alginates are anionic linear PSs found in brown seaweeds, usually from *Laminaria* sp. and *Ascophyllum nodosum*. Alginate has been reported as being able to form edible films. Indeed, due to its excellent stabilizing and thickening properties, it is commonly used in food products and medicine [71,73,74].

**Figure 5:**
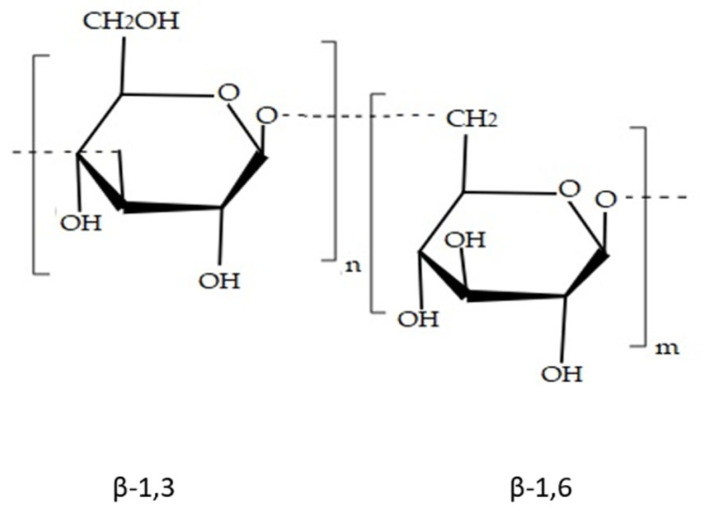
Chemical structure of laminarin.

Laminarin is a linear polysaccharide made up of β-(1,3)-glucan with β-(1,6)-branches with repeated units (Figure 5). It is used in the food industry and biomedicine because of its various bio-functional properties when modified trough chemical processes [75]. For example, laminarin has immunostimulatory effects that can activate macrophages, leading to the healing of wounds and antitumour properties [76]. An enhanced antioxidant activity is also observed when this laminarin is irradiated with gamma rays [77]. 

**Figure 6:**
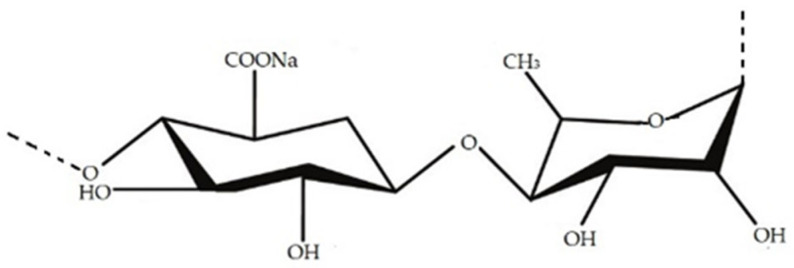
Chemical structure of ulvan.

Ulvan is a PS common in green algae. The ulvan backbone is most made up of α- and β-(1,4)-linked monosaccharides that can vary (rhamnose, xylose, glucuronic acid and iduronic acid), with characteristic repeating disaccharide units [78] (Figure 6). Even though the potential of ulvan needs to be better acknowledged, some studies already prove its potential in pharmaceutical and industrial sectors [62,79,80,81].

Polyphenols are compounds which contain one or more aromatic rings bearing hydroxyl groups, and they have been shown to exhibit antioxidant, antimicrobial, antidiabetic, anti-inflammatory and anticancer properties in in vitro and in vivo studies [82]. The antioxidant activity and redox potential of polyphenols allow them to reduce the reactive oxygen species (ROS) involved in several human disorders [83]. Antioxidant compounds extracted from seaweeds are already available on the market for their health benefits or for their ability to prolong food expiring [84].

**Figure 7:**
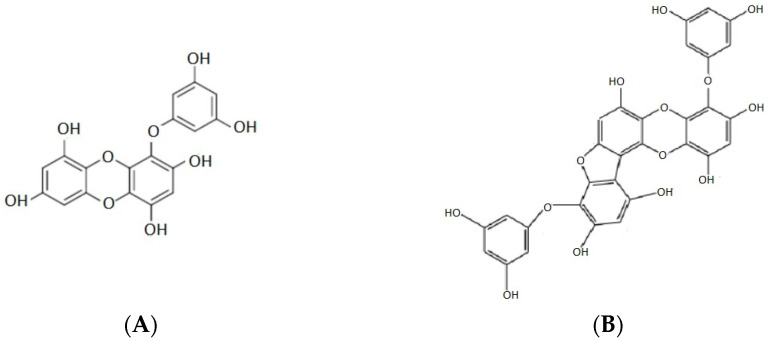
Chemical structure of eckol (**A**) and phlorofucofuroeckol A (**B**); phlorotannins isolated from *Ecklonia cava*.

Among several classes of algal polyphenols, phlorotannins are the most known as they have shown pharmacologically potential. According to the variety of structural linkages between phloroglucinol units (1,3,5-trihydroxybenzene) and the number of hydroxyl groups present, phlorotannins are divided into six specific groups: phlorethols, fuhalols, fucols, fucophlorethols, eckols and carmalols [85] (Figure 7). 

Diverse research focuses on the isolation and properties of phlorotannins, as many of them possess antimicrobial [86], antioxidant [87], anti-HIV [88], antiproliferative [89], anticancer [90], anti-inflammatory, antidiabetes [91], anti-Alzheimer diseases, antihypertensive [92] and radioprotective [93] properties. The presence of phlorotannins in nature is limited to brown algae and their number and functions depend on the condition of algae (size, age, type of algae), environment (light, water temperature, salinity, seasonality) and ecosystem (nutrient levels, magnitude of herbivory) [94].

A huge group of natural products commonly found in plant and marine algae are terpenoids. They are a unique class of algae secondary metabolites comprising terpenes attached to an additional functional group, usually containing oxygen. Several studies suggest that these compounds may be employed for treatment of cancer cells as well as for mammary, skin, lung, forestomach, colon, pancreatic and prostate carcinomas cells, being open to development of new cancer therapies [95,96]. Terpenoids are commonly classified as monoterpenoids, sesquiterpenes, diterpenes, sesterterpenes based on their chemical structure, which may vary from species to species.

A literature revision performed by Hannan et al. [97] updated information on the health effects of seaweed phytosterols, compounds with high reputation in therapeutic activity for their cholesterol-lowering potential [98]. Moreover, phytosterols of marine algae, particularly fucosterol, have been investigated for health benefits, including anti-diabetes, anti-obesity, anti-Alzheimer’s, antiaging, anticancer and hepatoprotection, among many others.

Nevertheless, for edible algae, it is important to consider heavy metals present in their biomass. Heavy metals discharged in aquatic environments can damage marine species and could be dangerous for human health through their accumulative behaviour and toxicity [99]. Toxic elements such as Al, Pb, Hg, Cd and As can be damaging even at low levels when ingested over a long period of time. The same is true for an excessive intake of essential metals [100].

The quantity of toxic metals as Al, Cd, Pb and Hg has been determined in European and Asian edible seaweeds. Al was the major toxic metal found (57.5 mg Al/kg dry weight), followed by Pb (0.40 mg/kg dw), with the highest average concentration found in seaweed salad from Asia (*Undaria pinnatifida*, *Laminaria ochroleuca*, *Himanthalia elongata*). The highest average concentration of Hg was recorded in *Laminaria ochroleuca* (the European kombu). As for Cd, it is worth mentioning the concentration recorded in *Undaria pinnatifida* (wakame) from Asia of 0.59 mg/kg dw [101], probably due to the high levels of industry in the Asian countries.

The regulation of the European Commission (EC) n° 488/2014 [102] defined the maximum limit only for Cd on algae (3 mg/kg), but no limits are defined for Al and Pb in seaweeds [103].

However, French legislation sets maximum levels for Cd (0.5 mg/kg dw) and for Pb (5 mg/kg dw) in edible French algae [101]. Roleda et al. [104] detected the presence of heavy metals in *Saccharina latissima* and *Alaria esculenta* (brown algae) and *Palmaria palmata* (red alga) from natural population and aquaculture of North Europe. Data suggest a concentration of heavy metals below the upper limits set by the French recommendation and the EU Commission Regulation on contaminants in food products, identifying these algae as not harmful for human consumption.

However, there is a need for guidelines regarding the limit and quality of seaweed consumption, with a general European regulation that establishes the maximal concentration of heavy metals in seaweeds, to not exceed seaweed consumption and preserve our health.

**Table 1:** Main biological properties and industrial applications of seaweed’s bioactive compounds.

Class of Seaweed Bioactive Compounds		Application and Properties	Principal Source	Reference
Polysaccharides	Alginate	Used as stabilizer and thickening agent in food products and medicine	Brown seaweed (*Laminaria* sp., *Ascophyllum nodosum*)	[71,73,74]
	Fucoidan	Antiproliferative, Antimicrobial and Antiviral activity Anticoagulant activity Antidiabetic activity	Brown seaweed (*Undaria pinnatifida*, *Fucus* sp.)	[105,106,107,108,109,110,111,112,113,114]
	Laminarin	Used in food industry and biomedicine because of its nutraceutical properties; immunostimulatory, antitumour and antioxidant activity	Brown seaweed (*Laminaria* sp.)	[75,76,77,115]
	Agar	Used in food products and pharmaceutical field as jellifiers, stabilisers, thickeners and emulsifiers	Red seaweed (*Gracilaria* sp.)	[116,117,118,119,120]
	Carrageenan		Red seaweed (*Gigartina* sp., *Chondrus* sp.)	[116,117,121,122,123]
	Porphyran	Anti-inflammatory, antioxidant, antihyperlipidemic and anticancer activities	Red seaweed (*Porphyra* sp.)	[59,60,61]
	Ulvan	Immunostimulatory, antitumoural, antiviral activities	Green seaweed (*Ulva* sp.)	[62,79,80,81,124]
Polyphenols	Phlorotannin	antimicrobial, antioxidant, antiviral, anticancer, anti-inflammatory, antidiabetic properties	Brown seaweed (*Ecklonia* sp., *Eisenia* sp., *Laminaria* sp., *Undaria pinnafitida*)	[86,87,88,90,91,125]

## 3. Seaweeds in Pharmaceutical Studies and Applications

Due to the extensive exploitation of terrestrial biological resources, it is fundamental to find new resources for human health research and drug development. Along the years, the research on marine drugs has obtained important achievements; several studies assess in vitro and in vivo tests to explore biological compounds of marine organisms, with the aim of evaluating their mechanisms of action and exploiting them for pharmaceutical purposes. On this ground, research holds on to this new branch of pharmacy, the marine pharmacology, which involves the integration of multiple disciplines and requires the comprehensive understanding and application of multiple knowledge [126]. 

Before pharmacological tests, it is necessary to verify the potential of seaweed bioactive compounds. Through standardization and quality control of parameters, it is possible to have a clear spectrum of the components included in seaweeds, which will be correctly identified and tested. Such studies will ensure the identity, quality and efficacy of biological compounds [127]. Examples of pharmacognostic analysis are reported for *Chaetomorpha antennina*, *Ulva lactuca* [128], *Sargassum wightii* [129] and *Sargassum cinereum* [130]. 

The study of pharmacokinetics profiling of active molecules is an essential step in drug development. Pharmacokinetics studies how a drug acts after administration via the processes of absorption, distribution, metabolism and excretion. The understanding of the pharmacokinetics of marine-derived polysaccharides has led to their potential use in pharmaceutical formulations [131].

Although the number of reports about new marine-derived compounds has increased, only a few have been reported a pharmacokinetic pathway. For example, Pozharitskaya et al. [132] reported the pharmacokinetics of fucoidan from *Fucus vesiculosus* after oral administration to rats. Other examples are given by Arunkumar et al. [133], who reported on the pharmacokinetic profiling of in vitro seaweed sulphated polysaccharides against *Salmonella typhi*. Shannon et al., [134] have shown seaweed’s bioactive compounds after oral administration acts as prebiotics and positively modulates the gut microbiota. Ventura et al. [135] reported on pharmacokinetic studies that evidence safety after *Fucus vesiculosus* polysaccharides oral absorption. 

Topical applications have several advantages compared to the pathway of oral administration: the pharmacokinetics are based on skin absorption; this avoids extensive first-pass metabolism and provides direct access and localization at the site of action. Topical applications are usually well-tolerated and can be an alternative for patients who cannot use other administration routes [136,137]. Pozharitskaya et al. [138] investigated the pharmacokinetics after topical application of fucoidans from *Fucus vesiculosus*. Data suggest that a significant amount of fucoidan-based drug was retained in the skin after the topical dose and accumulated in the striated muscle. Single doses (50–150 mg/kg) and multiple doses (100 mg/kg over five days) of topical application caused no signs of dryness, erythema, hemorrhage, edema or erosion/excoriation in skin rats. The kinetics of topical application of fucoidan have been also investigated by Obluchinskaya et al. [139] to evaluate the anti-inflammatory activity of fucoidan extracted from *Fucus vesiculosus* in carrageenan-induced rat paw edema. The fucoidan-based cream dose-dependently inhibited paw edema after topical application, with an efficacy at higher doses equal to diclofenac gel, a synthetic anti-inflammatory drug used to treat pain and inflammatory diseases. Results suggest that fucoidan release was controlled by drug diffusion into the skin, thus, the topical application of fucoidan-based cream might be an effective method to treat inflammation and skin diseases.

Therefore, a deep characterization of pharmacokinetics can give us useful information to better understand the molecular basis behind the pharmacological activity, to correct doses and treatment, obtaining more selective drug applications [131]. In Table 2, several cases of in vitro and in vivo studies are reported to confirm the potential of seaweeds bioactive compounds in medicine and pharmaceutical applications.

### 3.1. Phylum Ochrophyta, Class Phaeophyceae

Phaeophyceae are predominantly brown in colour due to their content of carotenoid fucoxanthins. These algae are recognized as an important source of bioactive compounds and other elements beneficial for human health. Fucoxanthin is an orange-coloured xanthophyll pigment [202,203] found in high content in Phaeophyceae, Haptophyta, Bacillariophyceae, Chrysophyceae and, to a lesser extent, in Rhodophyta, Raphidophyceae and Dinophyta. 

Those pigments not only give the algae their peculiar colour, but they also exhibit several biological activities such as anti-inflammatory [204,205], anti-obesity [206,207], antiangiogenic [208] and anticancer properties which can be exploited for pharmaceutical purposes. In vivo and in vitro assays showed inhibition of tumour growth in lung cancer due to fucoxanthin isolated from *Laminaria japonica* [140], while fucoxanthin isolated from the marine alga *Ishige okamurae* inhibited B16-F10 melanoma cells implanted in albino mice [168]. Chung et al. [141] isolated fucoxanthins from the brown alga *Laminaria japonica* with the intent to evaluate their effect on in vitro metastasis models of B16-F10 melanoma cells. Tests showed a significant reduction in metastasis; the further step was testing fucoxanthins in vivo on lung metastasis. In conclusion, Ching et al. [141] demonstrated a significant inhibition in lung cancer cells’ growth, proving that fucoxanthins might be value in preventing cancer metastasis [141]. Atya et al. [142] isolated fucoxanthin from *Colpomenia sinuosa* and *Sargassum prismaticum* to evaluate in vitro anticancer activity and in vivo antioxidant activity. HCT-116 (colon adenocarcinoma), MCF-7 (breast adenocarcinoma) and HepG-2 (liver adenocarcinoma) cell lines showed a reduction in growth, while the in vivo tests evaluated the hepatoprotective ability of fucoxanthin in mice. The results were positive, as the antioxidant and anti-inflammatory properties of the extract protected the hepatocytes from inflammation and membrane damage in mice [142]. Wang et al. [143] investigated the cancer cell growth inhibition of fucoxanthins extract from *Undaria pinnatifida*. Among the cancer cell lines investigated, melanoma Malme-3M and cervix squamous SiHa cells carcinoma showed major growth inhibition [143]. Although in vitro results are positive, it is necessary to improve the studies performing in vivo tests. The correlation between antioxidant activity and total phenolic content or fucoxanthin has been as well demonstrated by Yan et al. [209]. Results showed higher antioxidant activity in *Sargassum horneri* and *Cystoseira hakodatensis* compared to *Eisenia bicyclis*, *Kjellmaniella crassifolia* and *Alaria crassifolia*. This could be explained by the higher total phenolic content and fucoxanthin in *Sargassum horneri* and *Cystoseira hakodatensis* [210].

Among PSs, fucoidans have exhibited several pharmacological properties. The structure and composition of fucoidans vary among different species of brown algae, thus the beneficial effects they provide also vary [211]. Several fucoidans express antiproliferative activity against cancer cells [105]. Boo et al. individuate anticancer activities in fucoidan from *Undaria pinnatifida* against human lung adenocarcinoma cell line A549 [144]. Shibata et al. [106] evaluated the action of fucoidans extracted from *Cladosiphon okamuranus*, a type of edible algae, during *Helicobacter pylori* infection, confirming the antimicrobial activity. The mechanism of defence is due to the inhibition of urease enzyme avoiding the *Helicobacter pylori* adhesion to the gastric mucosa. Palanisamy et al. [107] isolated fucoidan from *Spatoglossum asperum* to detect antimicrobial activity against *Aeromonas hydrophila* (Gram-negative bacteria) using agar bioassay. In vitro antioxidant activity was evaluated by 2,2-diphenyl-1-picrylhydrazyl (DPPH) radical scavenging activities, resulting in a reduced power and total antioxidant activities. Results showed that fucoidan extracts exhibit antioxidant properties in a dose-dependent manner, as well as for antibacterial properties [107]. Antibacterial properties were also investigated with crude fucoidans and purified fucoidans from the brown algae *Fucus vesiculosus*. For both fucoidan preparations, a bacteriostatic effect was observed on *Escherichia coli*, *Staphylococcus epidermidis*, *Staphylococcus aureus* and *Bacillus licheniformis*, with *Escherichia coli* being the most sensitive to each of the fucoidans. Results showed that the purification of fucoidan lead to a change of monosaccharide composition with a decrease in the sulphate and uronic acid contents, that generate a decrease in its antimicrobial activity [108]. In another study performed by Liu et al. [109], crude fucoidans from *Laminaria japonica* exhibited no antibacterial activity, while depolymerized compounds possessed good antibacterial activity both against *Escherichia coli* and *Staphylococcus aureus*. Depolymerized fucoidans combine with the membrane proteins and cause a membrane-disrupting effect, leading to the collapse of membrane structure and eventually resulted in cell death. Therefore, further studies that correlate antimicrobial activity and structure of fucoidans must be performed and better elucidated. 

Antiviral effects of PSs have been assessed against herpes virus strains. Native fucoidan from *Fucus evanescens* and its derivative were tested against *Herpes Simplex Virus* type 1 (HSV-1), *Herpes Simplex Virus* type 2 (HSV-2), enterovirus (ECHO-1) and human immunodeficiency virus (HIV-1). The investigation of both native fucoidan with nonregular structure and derivates can give us the answer of which structural fragment of these unique polysaccharides is important against viruses. In vitro assays were performed in African green monkey kidney (Vero) cells; for in vivo tests, intravaginal HSV-2 infection was induced in female mice. In vitro results showed the ability of both fucoidans to increase the resistance to virus, directly affecting the cell and inhibiting early stage of virus replication. Comparative analysis of antiviral activities of crude and fragmented fucoidans showed that crude fucoidans more effectively inhibits the replication of both types of HSV. Antiviral activity against ECHO-1 and HIV-1 has been detected but is lower than HSV strains. Furthermore, in vivo studies showed that intraperitoneal administration of fucoidans in mice protected the animals from lethal intravaginal HSV-2 infection [110]. The antiviral effect against HSV-1 and HVS-2 was also individuated from sulphated polysaccharide isolated from *Sargassum patens* [145,146], where it has been detected an increase of anti-HSV activity with increasing sulphate ester content of polysaccharides [212]. These results suggest the feasibility of inhibiting HSV infection with seaweeds polysaccharide having specific structure against the virus. 

Pozharitskaya et al. [111] elucidated the anti-coagulant bioactivities of the high molecular weight of fucoidan from *Fucus vesiculosus* in several in vitro model, which has been confirmed by de Azevedo et al. [112], as well. The anticoagulant activity of fucoidan has been also detected in in vivo assay, where human volunteers consumed 3 g for 12 days of oral administration of fucoidan from *Undaria pinnatifida* [113].

Fucoidans have been discovered being active against diabetes issues. Fucoidans isolated from *Undaria pinnatifida* were investigated against three starch hydrolysing enzymes, α-amylase, α-glucosidase and amyloglucosidase. It was demonstrated that while the fucoidan extract exhibited significant inhibitory effects against all the three starch hydrolases, it exhibited significantly stronger inhibitory effects against α-glucosidase, suggesting fucoidans from *Undaria pinnatifida* as an antidiabetic agent [114]. Jia et al. [169] investigated PSs structural characterization and antidiabetic activity from *Sargassum fusiforme* and *Macrocystis pyrifera* in high-fat diet and streptozotocin-induced diabetic rats. Oral administration of PSs restrained loss of weight and increased water intake, significantly controlling the increase of levels of blood glucose, triglyceride and total cholesterol in diabetic rats. Therefore, polysaccharides from brown seaweeds could be promising candidates as natural medicines and functional foods for the improvement of diabetes problems. 

Polyphenols are other powerful compounds with interesting biological activities. Extracted from *Padina australis* found to inhibit microbial growth by damaging the cytoplasmic membrane and destroying the cell bacteria. Phenols and flavonoids attack the phosphate group leading to the breakdown of phospholipid molecules of the cell bacteria into carboxylic acid, glycerol and phosphoric acid. As a result, the growth of bacteria is retarded and they eventually die [147]. Chkhikvishvili and Ramazanov [147] already demonstrated the bactericidal potential of phenolic extracted from *Padina australis* on *Bacillus cereus*; Kumar et al. [148], through in vitro bioassay, showed inhibition against beta-lactamase negative *Escherichia coli* ATCC 25922, *Pseudomonas aeruginosa, Staphylococcus aureus* and *Bacillus cereus* by phenolic extracts of *Padina australis*, revealing that this species possess narrow-spectrum antibacterial activity. 

Seaweeds have been primarily studied for their phloroglucinol based polyphenols, called phlorotannins. Among marine brown algae, phlorotannins extracted from *Ecklonia cava*, *Ecklonia stolonifera*, *Ecklonia kurome*, *Eisenia bicyclis*, *Sargassum thunbergii*, *Hizikia fusiformis*, *Undaria pinnatifida* and *Laminaria japonica* have been reported to exhibit health beneficial activities [125]. 

Nagayama et al. [86] examined the bactericidal effects of crude and purified phlorotannins from the brown alga *Ecklonia kurome* on pathogenic bacteria. Phlorotannins showed bactericidal activity against *Staphylococcus aureus*, *Streptococcus pyogenes*, *Bacillus cereus*, *Campylobacter fetus*, *Campylobacter jejuni*, *Escherichia coli*, *Salmonella enteritidis*, *Salmonella typhimurium* and *Vibrio parahaemolyticus*. Moreover, the effect post administration of phlorotannins on male and female mice was investigated to confirm the safety of phlorotannins for mammals. The experiment proves the antibacterial activity of phlorotannins from *Ecklonia kurome*. These results may suggest the addition of these compounds in food product or to develop drug with antibacterial activity with no harmful effects on our organisms. Lee et al. [162,163] analysed the antimicrobial activity of extract from *Ecklonia stolonifera*. It showed that dieckol purely isolated from *Ecklonia stolonifera* evidenced the antibacterial activity against methicillin-resistant *Staphylococcus aureus* and methicillin-susceptible *Staphylococcus aureus*, with MIC values ranging from 32 to 64 μg/mL.

The multiple activities of phlorotannins were already exploited in the traditional Korean medicine, where *Sargassum hemiphylum* was already used for the treatment of various allergic diseases. Na et al. [158] stated the significant contribution of *Sargassum hemiphylum* to the treatment of atopic allergic reactions, such as atopic dermatitis. Dioxinodehydroeckol (DHE) and phlorofucofuroeckol A (PFF-A) isolated from *Ecklonia stolonifera* contribute to attenuate allergic reactions and might be a promising candidate for the design of novel inhibitor allergic reaction [164]. In vivo assay on rats fed with *Eisenia arborea* showed inhibition of IgE and anti-degranulation of histamine, suggesting that *Eisenia arborea* might possess anti-allergic effects [170].

The antioxidant activity of phlorotannins has been detected in brown seaweeds to treat neurodegenerative diseases such as Alzheimer’s. It has been proved that the inhibition of AChE enzyme, which catalyses the breakdown of ACh, is a useful therapeutic approach for the symptomatic treatment of Alzheimer’s disease. The anticholinesterase activity of seaweed extracts has been tested against acetylcholinesterase (AChE) and butyryl cholinesterase (BChE), which are the main enzymes of Alzheimer’s disease [213]. Among the tested seaweeds, *Fucus spiralis*, *Bifurcaria bifurcata* and *Cystoseira stricta* had potential anticholinesterase activity, which could be used in the future as therapeutic agents for Alzheimer’s disease. Phlorotannins of the South African seaweed *Dictyota humifusa* were extracted to test acetylcholinesterase inhibitory activity; results showed that the extracts were effective at inhibiting AChE [159]. Moreover, the inhibitory effect on AChE and BChE activities measured by Custodio et al. [149] suggests a possible therapeutic value of phenols present in *Cystoseira tamariscifolia* and *Cystoseira nodicaulis*, as they are reported as potential anticholinesterase inhibitors. Further screening of AChE and BChE inhibitory activity on ethanol extracts of different seaweeds showed that extracts from *Cystoseira usneoides* and *Fucus spiralis* were potent inhibitors [214]. It has also been found that phenol found in *Ecklonia maxima*, *Ecklonia stolonifera* and *Ishige okamurae* were also able to inhibit AChE [91,150], as well as dieckol and phlorofucofuroeckol, two phlorotannins found in brown algae *Eisenia* sp. and *Ecklonia* sp. [165].

Phlorotannin-rich fractions extracted from *Cystoseira sedoides* (PHT-SED), *Cladostephus spongeosis* (PHT-CLAD) and *Padina pavonica* displayed in vivo antioxidant activity among all three species, with higher activity for *Cystoseira sedoides*. The study demonstrated that phlorotannins have a strong antioxidant activity towards radicals, showed by the anti-inflammatory assays resulting in a reduction of paw edema and ear thickness in a dose-dependent way in mice tested. The anti-inflammatory potential of phlorotannins is also due to the inhibition of oxidative stress by decreasing the production of malondialdehyde (MDA), which is manifested with an increase in free radicals [215]. Kim and Kim [157] reported an anti-inflammatory effect from phloroglucinol, a monomer of phlorotannins, derived from *Ecklonia cava*. The antioxidant mechanism of phloroglucinol can be attributed to three hydroxyl groups existed in phloroglucinol that can react with ROS.

Properties expressed by phlorotannins are multiple; it has been reported that eckol isolated from the brown algae *Ecklonia cava* possess potent antiproliferative activity against human breast cancer cells MCF-7. Moreover, dioxinodehydroeckol, a phloroglucinol derivative, can induce apoptosis through the NF-κB-dependent pathway. In vivo studies assess that dietary inclusion of brown algal polyphenols significantly reduced the tumour proliferation in the pre-tumour bearing mouse. These polyphenols prevent the tumour progression in vivo by inhibiting the activity of cyclooxygenase-2 and cell proliferation [89]. Phlorotannin extracts from *Fucus vesiculosus*, *Alaria esculenta*, *Ascophyllum nodosum*, *Laminaria japonica*, *Sargassum muticum* and *Bifurcaria bifurcata*, among others, were shown to dose-dependently reduce the cell proliferation of numerous tumour cell lines such as human fibroblast (HFF-1), gastric cancer cells (MKN-28), human colon cancer cells lines (HT-29 cells and Caco-2), human hepatoma (BEL-7402), mouse leukaemia (P388) and mouse teratocarcinoma (ATDC5) [151,152,153,154,155,156].

Phlorotannins may also be implied in cosmetics, as their compounds possess the ability to prevent the skin aging process due to the inhibition of hyaluronidase, an enzyme able to degrade the hyaluronic acid present in the extracellular matrix. Phlorotannins derivatives such as fucophloroethol, fucodiphloroethol, fucotriphloroethol, 7-phloroeckol, phlorofucofuroeckol and bieckol/dieckol extracted from *Cystoseira nodicaulis* exhibited hyaluronidase activity, becoming potential candidates for the production of antiaging creams [171]. Dieckol, eckol, bieckol, and phlorofucofuroeckol A extracted from *Eisenia bicyclis* and *Ecklonia kurome* also exhibited potent inhibition towards hyaluronidase [166]. 

Phlorotannins extracted from *Ecklonia cava* and *Ecklonia stolonifera* also provided photoprotection towards UV rays by reducing the cell damage caused by solar radiation, being good candidate for the development of sun creams protection [160,161]. 7-phloroeckol and dieckol isolated from the brown algae *Ecklonia cava* had higher inhibitory activity against tyrosinase, an enzyme linked with the melanin hyperpigmentation of skin. These compounds showed higher inhibitory activity than those of commercial inhibitors such as arbutin and kojic acid [161,167]. The inhibition of tyrosinase plays an important role in skin related disease, indeed tyrosinase inhibitors are already used as a whitening agent in cosmetics and clinical skin treatments [216].

### 3.2. Phylum Rhodophyta

Red algae favour intertidal and subtidal zones of rocky coasts, and many of them are located deeper than brown and green algae, in cold and temperate areas [217]. In the last decade many in vitro and in vivo experiments have confirmed the pharmaceutical potential of red algae extracts [218,219,220]. Phycoerythrins of red algae possess interesting biological activities; Sekar and Chandramohan [221] describe the antitumoural effect of those pigments in mouse tumour cells and human liver carcinoma cells. Other effects reported include antioxidant [33,222] and antidiabetic [222], making phycoerythrins a good alternative in marine pharmaceutical [32]. 

Red seaweeds are the sole source of certain valuable polysaccharides, namely agar and carrageenan. Their interesting biological properties may be applied in pharmaceutical and medical applications. 

Carrageenans differ from structure and type, and each show different biological effects [223]. The antiviral activity of carrageenan from *Gigartina skottsbergii* against intraperitoneal murine HSV-1 and HSV-2 infection has been proved [121]. Matsuhiro et al. [172] extracted polysaccharide from *Schizymenia binderi* to evaluate in vitro antiviral activity against HSV-1 and HSV-2 by a plaque reduction assay in Vero cells. Results express the inhibition of the strains, in a similar way that ɩ- and k-carrageenans isolated from *Gigartina skottsbergii* express their antiherpetic activity [121,176]. Talarico and Dalmonte [224] demonstrate λ-carrageenan and ι-carrageenan as potent inhibitors of dengue virus type 2 (DENV-2) and type 3 (DENV-3). *Pterocladia capillacea* and *Laurencia obtusa* were investigated for their antiviral activity against Hepatitis C Virus in vitro; hepatocellular carcinoma HepG2 cell and peripheral blood cells were infected with the virus. Results showed antiviral activity for both species, but polysaccharides from *Laurencia obtusa* were revealed to be the most inhibitor [10].

Seaweed polysaccharides possess promising antiviral properties regarding a broad spectrum of the activity, as well as the complex mode of action. Therefore, seaweeds represent an interesting candidate for further antiviral development, as well as for antimicrobial treatment. Kulshreshtha et al. [179] describe the combined effects of selected extracts of two red seaweeds, *Chondrus crispus* and *Sarcodiotheca gaudichaudii* with two well-used antibiotics (tetracycline and streptomycin) against *Salmonella Enteritidis*. Both in vitro and in vivo tests proved enhanced antimicrobial activity. In vivo assays were performed by ingestion of algal extracts by infected hens, while in vitro tests involved combination with seaweeds extracts and sub-lethal dose of tetracycline and streptomycin [177,178]. 

Antitumoral potential of carrageenans has been also widely evaluated. Carrageenans extract from the genera *Gigartina*, *Chondrus*, *Eucheuma* and *Iridaea* spp., which showed that κ-carrageenan significantly prevent in vitro growth of fibroblasts, HeLa cells and mammary cells [174]. ɩ/ε carrageenans extracted from red seaweed *Gigartina pistillata* demonstrate in vitro potential against colorectal cancer stem cells-like cells [175]. The anticancer effect of carrageenan varies based on the molecular weight of the compound; Khotimchenko et al. [225] reviewed several in vitro and in vivo assays regarding the antitumour potential of native carrageenans and oligocarrageenans. In most cases, oligocarrageenans show higher activity then native carrageenans.

Lins et al. [184] evaluated both in vivo and in vitro antitumoural effect of polysaccharides extracted from *Champia parvula*. In vivo tests showed the antitumoural effect with sarcoma 180 ascites tumour cells implanted subcutaneously into mice. Moreover, the cytotoxicity of these polysaccharides was tested in vitro against proliferation of HL-60 (human leukaemia), MDA-MB-435 (melanoma), SF-295 (brain), and HCT-8 (human colon) cell lines. The data demonstrate that polysaccharides from *Champia parvula* inhibited sarcoma 180 tumour growth in mice, but had no effects on human cells proliferation in vitro at the tested concentrations. Furthermore, when the mice were treated simultaneously with both the sulphated polysaccharide and the chemotherapeutic agent fluorouracil (5-FU), the tumour inhibition rate increased significantly [184]. This is a very interesting finding to improve the efficacy of anticancer therapy, developing efficient combination of chemotherapeutic drugs, whereas the side effects are reduced. Zhou et al. [123] prove the antitumoural activity of extracts from *Chondrus ocellatus* on H-22 tumour cells implanted on mice. The ingestion of a mixture of ʎ-carrageenan and 5-FU results in an enhanced antitumour activity. 5-Fu is usually included in chemotherapy treatments due to their suppression of many tumour cells. Unfortunately, also useful cells (e.g., immune cells) are destroyed by this immunosuppressive. Thus, the implementation of natural substances in chemo-treatments may reduce the side effects [123,226,227]. Finding new natural sources with anticancer activities might give the possibility to develop efficient combination of chemotherapeutic drugs, where the side effects are reduced.

Carrageenans also possess anticoagulant activity, as Andrade et al. [173] described in their in vitro study. *Solieria filiformis* carrageenans were tested to detect anticoagulant effects. The extracts were assessed by both in vitro activated partial thromboplastin time (APTT) and prothrombin (PT) tests. Results showed a reduction and inhibition of thrombin and factor X in blood coagulation, making *Solieria filiformis* a good candidate for the development of anticoagulant natural drugs [173]. Although seaweeds extracts are not potent as heparin, other studies confirm the anticoagulant potential of carrageenans [228,229,230,231].

These peculiar compounds are already available in the market as potent food supplements. Moreover, their biological activity gives benefits to health. The ingestion of ι-carrageenan extracted from *Sarconema filiforme* [181] and κ-carrageenan extracted from *Kappaphycus alvarezii* [182] were investigated in vivo. Results showed that both carrageenans decreased body weight, systolic blood pressure, abdominal fat, liver fat and plasma total cholesterol concentrations in mice models. The assumption of polysaccharides derived from *Gracilaria lemaneiformis* showed as well anti-obesity activities in obese hamsters, with a decrease in the weight of adipose tissue, body and liver and lower plasma leptin, total cholesterol and triglyceride levels. Obesity increases cardiovascular risk by elevating blood sugar levels and lowering insulin concentration. The positive results given by red algae polysaccharides in decreasing blood glucose, improving glucose tolerance, increasing levels of C-peptide and liver glycogen, might suggest that these compounds might be useful for the development of anti-obesity and antidiabetic drugs [183,232]. 

Antioxidant activities have been investigated in extract from the Vietnamese red seaweed *Laurencia dendroidea* using DPPH, nitric oxide radical scavenging and metal chelating assays. The antidiabetic activity was tested in vivo on mice. The extract of *Laurencia dendroidea* showed strong α-glucosidase inhibitory and DPPH radical scavenging activities. Methanolic concentrations affected both α-glucosidase inhibitory and antioxidant activities. The seaweed extract was fractionated using different solvents (n-hexane, chloroform, ethyl acetate, butanol, and water); ethyl acetate fraction had the highest inhibitory activities against α-glucosidase inhibition and the strongest antioxidant activities. It also significantly reduced blood glucose level in diabetic mice compared with the diabetic control group [185].

Methanolic extract of the red seaweeds *Spyridia filamentosa*, *Grateloupia lithophila* and *Hypnea musciformis* was evaluated against α-amylase and α-glucosidase by spectrophotometric assays. The strongest inhibitor effect to α-amylase and α-glucosidase was shown by that crude methanolic extract of *Spyridia filamentosa*, which could have anti-diabetic potential through inhibition of α-amylase and α-glucosidase [180].

Carrageenan and agar are found to be used in food industry due to their capacity to form gels and increase viscosity of aqueous solutions [119,233,234,235]. Although, their interesting properties make agars suitable for pharmaceutical purposes. Extraction of agar-type polysaccharide from *Gracilaria dominguensis* possesses potent in vivo antitumour activity by inducing apoptosis and inhibiting the transplantation of Ehrlich ascites carcinoma in mice [118]. Indeed, agar oligosaccharides may be used as an alternative or complementary drug for people with diseases [236].

### 3.3. Phylum Chlorophyta

Chlorophyta possess high quantity of organic compounds that are interesting for pharmaceutical applications. For example, Ripol et al. [196] detected anti-inflammatory activities for five species of green seaweeds, such as *Chaetomorpha linum*, *Rhizoclonium riparium, Ulva intestinalis*, *Ulva lactuca* and *Ulva prolifera*. All species present inhibition to cyclooxygenase-2 (COX-2), the enzyme responsible for inflammation. Although, the screen of the compounds in the extract has not been performed, future research should focus on the extraction of the bioactive compounds from green macroalgae [196]. There are few studies on the anti-inflammatory activity of green seaweeds, ranging from in vitro assays to in vivo models. In vivo study performed by Bitencourt et al. [201] saw the treatment of mice with ulcerative colitis induced with methanolic extract of *Caulerpa mexicana*. As results show, the clinical signs observed amelioration in ulcerative colitis. The decreased level of cytokines observed could be associated with reduction in tissue damage found in the colon of the animals treated with algal extracts. Therefore, this extract appears promising for research on metabolites that may be helpful for anti-inflammatory treatment [201]. Different authors investigated the neuroprotective effects and anti-inflammatory activities of *Ulva conglobata* methanol extracts in hippocampal neuronal HT22 cells and mouse microglial BV2 cells. In this case, *Ulva conglobata* methanol extracts inhibited both iNOS, decreasing the production of free radicals and COX-2 expressions in microglia, as well as protecting hippocampal neurons against glutamate toxicity [198]. 

It is likely that the anti-inflammatory effects may be triggered by the alkaloid caulerpin or polysaccharides. During the in vivo assay performed by de Souza et al. [190], individuals of Swiss albino mice were pre-treated with caulerpin from *Caulerpa racemosa* briefly before the pain induction. Formalin-pain induction was performed with an injection of 20 μL of a 2.5% (*v*/*v*) solution of formalin in saline. Results indicate a high anti-inflammatory activity on mice [190]. Lucena et al. [191] tested the anti-inflammatory activity of caulerpin from *Caulerpa racemosa* to reduce leukocyte recruitment in vitro on zymosan-induced peritonitis model and in vivo on DSS-induced ulcerative colitis mice. The submitted doses of caulerpin were 40 and 4 mg/kg of body weight. Successfully, caulerpin extracts present positive results in anti-inflammatory potential both in vitro and in vivo experiments. The 4 mg/kg dose was able to attenuate weight loss and clinical signs, reduce colon size and decrease the levels of Th1 and Th17 pro-inflammatory cytokines. 

Zbakh et al. [195] recently investigated the anti-inflammatory and anticancer activity of dichloromethane and acetone/methanol extracts of the green alga *Codium decorticatum*. Results revealed that dichloromethane extract and acetone/methanol extract dramatically inhibited the expression of the pro-inflammatory cytokine in endothelial cells. Furthermore, dichloromethane extract showed cytotoxic activity against the HeLa cell line (cervix cancer cells) by inducing apoptosis at the highest concentrations, suggesting *Codium decorticatum* as potential candidate in therapeutic use for inflammatory diseases and cancer treatments. 

The polysaccharide ulvan from *Ulva lactuca* was proven to express anti-influenza A virus (IAV) activity [124], while the alkaloid caulerpin from *Caulerpa* sp. demonstrated antiviral effects on bovine viral diarrhea virus (BVDV) [187]. Viability test performed in Vero cells showed that caulerpin from *Caulerpa racemosa* has promising antiviral activity against HSV-1. The control test was performed with Aciclovir, common antiherpetic medication. In vitro tests showed that caulerpin inhibits the replication of the virus in early stages compared with Aciclovir [189]. The anti-HSV-1 activity has been confirmed in *Caulerpa brachypus*; different fractions of polysaccharides showed not only inhibition at the first stages of HSV-1 but also the penetration into host cells [192]. 

The activity of caulerpin was evaluated for its antimicrobial activity against *Mycobacterium tuberculosis* strain H37Rv. Results indicate that caulerpin may be useful as a lead compound for the development of novel anti-tuberculosis agents, thus, future studies may determine the mechanism of action for tuberculosis inhibition [188]. Agbaje-Daniels et al. [237] investigated the antimicrobial activity of five green seaweeds, namely *Ulva fasciata, Ulva lactuca, Chladophora vagabunda, Caulepa taxifolia, Chaetomorpha antennina* and *Chaetomorpha linum*. Extracts of each alga were tested against *Bacillus subtilis, Streptococcus pneumoniae*, *Streptococcus faecalis,* Gram negative species *Escherichia coli* (clinical and laboratory strain), *Escherichia coli* NCTC 10418, *Pseudomonas aeruginosa, Salmonella typhi* (clinical strain), *Salmonella typhi* NCTC 8385 *and Klebsiella pneumoniae*. It was observed that extracts of *Ulva fasciata* inhibited strains of *Staphylococcus aureus*, *Bacillus subtilis* and *Mycobacterium aurium* as well as the *Escherichia coli* strains tested, confirming the multidrug resistance of *Ulva fasciata* [238].

Results by Shanmughapriya et al. [239] report inhibition of the growth of *Staphylococcus aureus*, *Pseudomonas aeruginosa*, *Enterococcus feacalis* and *Escherichia coli* being tested with *Halimeda opuntia* extracts. Seaweeds belonging to *Halimeda* spp. have been investigated for several properties, as well as antioxidant, antibacterial and larvicidal activity [240]. Moreover, methanolic extracts from the macroalga *Halimeda tuna* showed antimicrobial activity on *Staphylococcus aureus*, *Salmonella typhimurium*, *Salmonella paratyphi*, *Klebsiella oxytoca*, *Escherichia coli* and antifungal activity against *Aspergillus niger*, *Aspergillus flavus*, *Alternaria alternaria*, *Candida albicans* and *Epidermophyton floccossum*, while isolated diterpenes exhibited antiviral activity on murine coronavirus strain A5Y [186,197].

Saeed et al. [194] tested chloroform extracts of *Ulva lactuca* and *Ulva fasciata* for antimicrobial activity against pathogenic bacteria (*Klebsiella pneumoniae* and *Proteus mirabilis*) and fungi (*Aspergillus flavus*, *Aspergillus fumingatus* and *Aspergillus niger*). The antibacterial activity was revealed for all strains; as the GC-MS chromatogram indicates, phenolic derivatives are the major constituents of the extracts responsible for the antimicrobial against tested pathogens. These extracts were also tested on human tumour cell lines: hepatocellular carcinoma (HepG2), mammary gland (MCF7), epithelioid Carcinoma (Hela) and human prostate cancer (PC3). *Ulva lactuca* extract showed strong cytotoxic activity against MCF-7, Hela cell lines, while *Ulva fasciata* extract had strong cytotoxic activity against PC3 and HepG2 cell lines.

An in vivo test performed injecting polysaccharides from *Ulva lactuca* into the caudal vein of rats proved inhibition of venous thrombus formation. The compound showed 56% reduction in the weight of the thrombus formed with polysaccharides (20 μg/g of rat weight), while the administration of heparin (1.5 μg/g of rat weight) reduced the thrombus weight of 92% [199]. This study evidences the anticoagulant potential of these sulphated PSs isolated from *Ulva lactuca*, making an exciting option for future investigation for anticoagulant drugs [199]. Therefore, *Ulva* spp. can be exploited for diverse purposes; extract of *Ulva rigida* showed anti-hyperglycaemic effect in experimental diabetes. Diabetes is usually accompanied by increased production of ROS and/or impaired antioxidant defence systems [241]. ROS can cause DNA strand breaks [242] and micronuclei, leading potentially serious consequences for the cell well-functioning [243,244]. Celikler et al. [200] performed in vivo assay to evaluate the potential genotoxic/antigenotoxic effect of *Ulva rigida* ethanolic extract injected in twenty-four male Wistar rats with induced diabetes mellitus. Extract from *Ulva rigida* showed genotoxic and/or cytotoxic effect, but also it is effective in reducing the chromosome damage induced by the diabetes [200].

Anticancer activity was evaluated also in fibroblast-like cells (L929) of C3H/HeJ mice connective tissue. In vitro tests with tumour cells and ulvan extracted from *Ulva lactuca* proved the cytotoxicity activity of this compound [81]. Antitumor in vitro tests have been conducted *Caulerpa racemosa* and *Caulerpa scalpelliformis* against human hepatoma cancer cells Huh-7 and human cervical cancer cells HeLa. The high contents of phenolic and flavonoid compounds present in *Caulerpa* spp. [245] allows these species to express several bioactivities, among this antitumoural activity [195]. 

Therefore, *Caulerpa* spp. and *Ulva* spp. are suggested as valuable source for functional food with a potential to be also used in pharmaceutical applications.

## 4. Use of Seaweeds in Traditional and Modern Pharmacology

The use of seaweeds for alimentary and medicinal purposes has been common since ancient times, especially in traditional medicine in Asian countries [246,247] even before the mechanisms of action of their compounds were acknowledged.

For example, the crude extract of the Chinese brown seaweed *Sargassum naozhouense* has been used to treat fever, infections, laryngitis and other ailments by the local population [248], while species of *Kappaphycus* and *Eucheuma* genera are used in Vietnamese medicine to reduce the occurrence of tumors, ulcers and headaches. *Sargassum* is used for treating iodine deficiency disorders such as goitre [249].

Valuable information regarding the use of *Sargassum* sp. was found in ancient transcript dated between the years 25–1061 AD. Most of the transcripts have been lost, although the information of the properties of *Sargassum* sp. have been re-examined and collected in Chinese medical books called “Compendium of Materia Medica”, written by Shizhen Li in 1578. The most ancient information about *Sargassum* regards the ability of this algae to treat thyroid related diseases such as goitre, but the Compendium also affirm that *Sargassum* sp. can soften hard lumps, dispel nodes, eliminate phlegm and induce urination in humans [250].

The information collected in the Compendium can be a valid starting point to evaluate which species of *Sargassum* were already involved to treat diseases and it provides the opportunity for the development of future research, as the lack of biochemical and pharmacological knowledge that ancient medicine had at the time [251].

Among *Sargassum* species, the traditional Chinese and Korean medicine adopted the use of *Sargassum pallidum*, *Sargassum confusum* to relieve and treat diseases given by goitre, scrofula, swelling and pain of testes, oedema due to retention of phlegm and morbid fluids. These brown algae have been investigated in modern Chinese medicine as well with positive outcomes; in fact, it has been proved that *Sargassum pallidum* and *Sargassum confusum* might also be used to treat arteriosclerosis, skin diseases, high blood pressure, hepatosplenomegaly, neurosis, angina pectoris, acute esophagitis and chronic bronchitis [252]. 

Recent research on those seaweeds suggested that *Sargassum* sp. may play a role as an immunomodulator, as their bioactive metabolites may inhibit thyroid growth induced by excessive iodine intake and improve immune function, which may be useful in treating Hashimoto’s thyroiditis [253]. *Sargassum thunbergii* and *Sargassum horneri* have been widely used as popular medicines and food ingredients in the southeast region of China, as well as for treating scrofula, goitre, sore throat, cough and phlegm stasis, angina pectoris, dropsy, dysuria and furuncle, giving the idea to be involved in the modern Chinese medical practice [253].

Other *Sargassum* sp. cited in the Compendium with similar effects are *Sargassum siliquastrum*, *Sargassum muticum*, *Sargassum hemiphyllum*, *Sargassum polycystum* and *Sargassum vachellianum* [250].

However, updated information regarding the treatment of thyroid related diseases (e.g., goitre) claims for *Sargassum* in traditional Chinese medicine has not been sufficiently researched yet.

However, with the development of new technologies, it has been possible to prove the beneficial effects of seaweeds on human health. In the last decades, in vitro and in vivo assays have been performed to prove antioxidant, antimicrobial, antiviral and antitumoural properties of seaweed extracts, with particular attention to the isolation and mechanism of action of each compound. Moreover, the use of seaweeds is sustainable and cheap, as they grow in every type of aquatic environment and are easily cultivated depending mainly on sunlight, aeration (natural or artificial) and seawater rich in nutrients. To overcome limitations related to common hydrocolloids extraction, which is time and energy consuming and involves chemical solvents, new extraction methodologies have been investigated. For example, the use of the deep eutectic solvents, as they can be prepared from natural compounds easily available. Smith et al. [254] classified deep eutectic natural solvents in four groups: (a) combination of organic salts and metal salts, (b) combination of organic salts and metal hydrates, (c) mixture of organic salts and compound being hydrogen bond donors and (d) combination of metal chlorides and compound being hydrogen bond donors. Their low costs, biopolymer dissolution ability, biodegradability, non-toxicity, polarity and recyclability make deep eutectic natural solvents a green alternative to replace organic solvents during seaweed’s polysaccharide extraction [255]. Indeed, these natural solvents have been successfully used in the extraction of polysaccharides from brown seaweeds [256,257,258,259] and red seaweeds [260], revealing the great potential of deep eutectic solvents in the extraction of seaweed biopolymers for industrial purposes. 

The versatility of properties exhibited by seaweeds compounds allows them to be involved in several biotechnological applications and give life to different kind of products. 

According to a study from Fortune Business Insights (https://www.fortunebusinessinsights.com/industry-reports/commercial-seaweed-market-100077, accessed on 20 November 2021), the global commercial seaweed market is projected to grow from $15.01 billion in 2021 to $24.92 billion in 2028. Over the past few years, the utilization of hydrocolloids as carrageenan, alginate and agar has been increased in food, pharmaceutical and other industries. 

Cargill, Inc. (agricultural products, Minnetonka, MN, USA) launched the Red Seaweed Promise™, a program specifically designed to address key sustainability challenges for the harvesting and cultivation of red seaweed needed to produce carrageenan, for various applications such as dairy, confectionery and personal care products. In the same way, since the 1960s, DowDuPont Inc. (chemicals, Midland, MI, USA) has been the pioneer in making seaweed harvesting as efficient and sustainable as possible, to develop new food sources. Other companies that invested in seaweed production cited in the report of Fortune Business Insights, are: Kerry Group PLC (food corporation, Tralee, Ireland), global leader in the development of taste and nutrition solutions for food, beverage and pharmaceutical markets; Acadian Seaplants (food-supply chain company, Dartmouth, Canada), active since 1981, the first industry to sell extracts of *Ascophyllum nodosum* and *Chondrus crispus* as unique seafood in the 1990s; Gelymar Industries (food, personal care and pharmaceutical products, Providencia, Chile), which has extracted hydrocolloids from seaweeds for food, pharmaceutical and personal care since 1990; Groupe Roullier (agriculture products, Saint-Malo, France), a French agribusiness conglomerate founded in 1959 that cultivates seaweeds for the development of animal and plant nutritional products; Corbion Chemicals Company (chemicals, Amsterdam, Netherlands), which released on the market AlgaPrime™ DHA, an algae-based omega-3 feed ingredients for aquaculture, production animals and companion animals; Ocean Harvest Technology Limited (animal feed, County Galway, Ireland), the company responsible for the development of OceanFeed™, an animal feed that helps animals to perform better in a natural way, as the feed ingredients are derived from seaweeds. OceanFeed™ has been also shown to support reduction of antibiotics and other synthetic feed ingredients.

It is important for companies that want to invest in the seaweed economy to adapt to a biology-based management. This means having sustainable approaches, adapting the harvesting and the production based on the life cycle and bioavailability of seaweeds. Moreover, it is necessary to empower seaweed producers, give them training and specific tools that will improve the yields of seaweeds, and have scientific consultants to detect the biological properties of seaweeds. 

## 5. Main Conclusions of the Pharmaceutical Potential of Marine Macroalgae and Perspectives

The scope of the present paper was to assess the potential of seaweed as a novel source for marine pharmaceuticals, which aims to develop novel drugs with compounds of natural origin and to diminish collateral effects due to synthetic compounds. Nowadays, the access to essential medicines is a luxury that only less than 50% of the world population has, due to the high price of medicines and the low purchasing power of the population of countries such as Africa and Asia [261].

To overcome health issues, the first line of defence of this population is the traditional medicine that involves the use of natural elements such as plants, herbs and seaweeds to treat diseases and disorders [262,263]. Therefore, the use of traditional medicine can be coupled with the modern medicine, as is happening nowadays in developed countries such as Canada, France and Australia [262]. However, seaweeds used in traditional medicine have a lack of proper biochemical characterization of the extracts, which can lead to dangerous outcomes, for example the insurgence of allergies or the assimilation of toxic compounds that can damage our organisms. There has been a high number of studies conducted about seaweeds’ compounds and extracts; nevertheless, further studies should be performed to go further and discover novel molecules to include in several biotechnological applications, thus directly and indirectly improving human welfare.

## Figures and Tables

**Table 2:** Pre-clinical study cases to demonstrate the potential pharmacological activity of brown, red and green seaweeds.

Species	Seaweed Compound	Pre-Clinical Study	Pharmaceutical Property	Reference
Phylum Ochrophyta, Class Phaeophyceae				
*Laminaria japonica*	Fucoxanthins	In vitro	Antitumoral activity on lung cancer cells	[140,141]
*Colpomenia sinuosa, Sargassum prismaticum*			Antitumoral activity on HCT-116, MCF-7, HepG-2 cells	[142]
*Undaria pinnatifida*			Antitumoral activity on Malme-3M, SiHa cells	[143]
*Undaria pinnatifida*	Fucoidans		Antitumoral activity on A549 cells	[144]
			Antidiabetic activity	[114]
*Cladosiphon okamuranus*			Antimicrobial activity against *Helicobacter pylori*	[106]
*Spatoglossum asperum*			Antimicrobial activity against *Aeromonas hydrophila*	[107]
*Fucus vesiculosus*			Antimicrobial activity against *Escherichia coli*, *Staphylococcus epidermidis*, *S. aureus*, *Bacillus licheniformis*	[108]
			Anticoagulant activity	[111,112]
*Laminaria japonica*			Antimicrobial activity against *Escherichia coli, Staphylococcus aureus*	[109]
*Fucus evanescens*			Antiviral activity against HSV-1, HSV-2, ECHO-1, HIV-1	[110]
*Sargassum patens*	Sulphate PSs		Antiviral activity against HSV-1, HVS-2	[145,146]
*Padina australis*	Phenols		Antimicrobial activity against *Bacillus cereus*, *Escherichia coli*, *Pseudomonas aeruginosa, Staphylococcus aureus*	[147,148]
*Cystoseira tamariscifolia, C. nodicaulis*			AChE-inhibitory activity	[149]
*Ecklonia maxima, E. stolonifera, Ishige okamurae*			AChE-inhibitory activity	[91,150]
*Fucus vesiculosus, Alaria esculenta, Ascophyllum nodosum, Laminaria, japonica, Sargassum muticum, Bifurcaria bifurcata*	Phlorotannins		Antitumoral activity on HFF-1, MKN-28, HT-29, Caco-2, BEL-7402, P388, ATDC5 cells	[151,152,153,154,155,156]
*Ecklonia cava*			Anti-inflammatory activity	[157]
*Sargassum hemiphylum*			Treatment of atopic dermatitis	[158]
*Dictyota humifusa*			AChE-inhibitory activity	[159]
*Sargassum hemiphylum*			Treatment of atopic dermatitis	[158]
*Ecklonia cava, E. stolonifera*			UV photoprotection	[160,161]
*Ecklonia kurome*	Phlorotannins		Antimicrobial activity against *Staphylococcus aureus*, *S. pyogenes*, *Bacillus cereus*, *Campylobacter fetus*, *C. jejuni*, *Escherichia coli*, *Salmonella enteritidis*, *S. typhimurium*, *Vibrio parahaemolyticus*	[86]
*Ecklonia stolonifera*	Phlorotannins (dieckol)		Antimicrobial activity against *Staphylococcus aureus*	[162,163]
	Phlorotannins (DHE, PFF-A)		Antiallergic effects	[164]
*Eisenia* sp., *Ecklonia* sp.	Phlorotannins (dieckol, PFFA)		AChE-inhibitory activity	[165]
*Eisenia bicyclis, Ecklonia kurome*	Phlorotannins (PFFA, dieckol, eckol, bieckol)		Hyaluronidase-inhibition activity	[166]
*Ecklonia cava*	Phlorotannins (7-phloroeckol, dieckol)		UV photoprotection in B16F10 melanoma cells	[167]
*Laminaria japonica*	Fucoxanthins	In vivo	Antitumoral activity on lung cancer cells	[140,141]
*Ishige okamurae*			Antitumoral activity on B16-F10 cells	[168]
*Colpomenia sinuosa, Sargassum prismaticum*			Antioxidant activity and hepatoprotective ability	[142]
*Fucus vesiculosus*	Fucoidans		Anticoagulant activity	[113]
*Fucus evanescens*			Antiviral activity against HSV-1, HSV-2, ECHO-1, HIV-1	[110]
*Sargassum fusiforme, Macrocystis pyrifera*	Sulphate PSs		Antidiabetic activity	[169]
*Ecklonia cava*	Phlorotannins		Antiproliferative activity on MCF-7 cells	[89]
*Eisenia arborea*			Antiallergic effects	[170]
*Cystoseira nodicaulis*			Hyaluronidase-inhibition activity	[171]
Phylum Rhodophyta				
*Schizymenia binderi*	Carrageenans	In vitro	Antiviral activity against HSV-1, HSV-2	[172]
*Solieria filiformis*			Anticoagulant activity	[173]
*Gigartina* sp., *Chondrus* sp., *Eucheuma* sp., *Iridaea* sp.	κ-carrageenans		Antiproliferative activity on HeLa cells, mammary cells fibroblasts	[174]
*Gigartina pistillata*	ɩ/ε-carrageenans		Antitumoral activity on colorectal cancer stem cells	[175]
*Gigartina skottsbergii*	ɩ- and k-carrageenans		Antiviral activity against HSV-1, HSV-2	[121,176]
*Chondrus crispus, Sarcodiotheca gaudichaudii*	Polysaccharides		Antimicrobial activity against *Salmonella Enteritidis*	[177,178,179]
*Pterocladia capillacea, Laurencia obtusa*	Sulphate PSs		Antiviral activity against HCV	[10]
*Spyridia filamentosa*	Methanolic extract		Antidiabetic activity	[180]
*Chondrus ocellatus*	ʎ-carrageenans	In vivo	Antitumoral activity on H-22 cells	[123]
*Sarconema filiforme*	ι-carrageenans		Anti-obesity activity	[181]
*Kappaphycus alvarezii*	k-carrageenans		Anti-obesity activity	[182]
*Gracilaria dominguensis*	Agar-type PSs		Anticancer activity on EAC cells	[118]
*Chondrus crispus, Sarcodiotheca gaudichaudii*	Polysaccharides		Antimicrobial activity against *Salmonella Enteritidis*	[177,178,179]
*Gracilaria lemaneiformis*	Sulphate PSs		Anti-obesity, antidiabetic activities	[183]
*Champia parvula*			Antitumoral activity on sarcoma 180 ascites cells	[184]
*Laurencia dendroidea*	Ethyl-acetate extract		Antidiabetic activity	[185]
Phylum Chlorophyta				
*Ulva lactuca*	Ulvan	In vitro	Antitumoral activity on L929 cells	[81]
			Antiviral activity against IAV	[124]
*Halimeda tuna*	Dipertene		Antiviral activity against coronavirus strain A5Y	[186]
*Caulerpa* sp.	Caulerpin		Antiviral activity against BVDV	[187]
			Antimicrobial activity against *Mycobacterium tuberculosis*	[188]
*Caulerpa racemosa*			Antiviral activity against HSV-1	[189]
			Anti-inflammatory activity	[190,191]
*Caulerpa brachypus*			Antiviral activity against HSV-1	[192]
*Caulerpa racemosa, C. scalpelliformis*	Phenols		Antiproliferative activity on Huh-7, HeLa cells	[193]
*Ulvs fasciata*			Antiproliferative activity on PC3, HepG2 cells	[194]
*Ulva lactuca*			Antiproliferative activity on MCF-7, HeLa cells	[194]
*Ulva lactuca, U. fasciata*			Antimicrobial activity against *Klebsiella pneumoniae, Proteus mirabilis*; antifungal activity against *Aspergillus flavus*, *A. s fumingatus*, *A. niger*	[194]
*Codium decorticatum*	Extracts		Anti-inflammatory, anticancer activities	[195]
*Chaetomorpha linum*, *Rhizoclonium riparium, Ulva intestinalis*, *U. lactuca, U. prolifera*			Anti-inflammatory activity	[196]
*Halimeda tuna*	Methanolic extracts		Antimicrobial activity against *Staphylococcus aureus*, *Salmonella typhimurium*, *S. paratyphi*, *Klebsiella oxytoca*, *Escherichia coli*; antifungal activity against *Aspergillus niger*, *A. flavus*, *Alternaria alternaria*, *Candida albicans*, *Epidermophyton floccossum*,	[197]
*Ulva conglobata*			Antioxidant activity	[198]
*Caulerpa racemosa*	Caulerpin	In vivo	Anti-inflammatory activity	[190,191]
*Ulva lactuca*	Sulphated PSs		Anticoagulant activity	[199]
*Ulva rigida*	Ethanolic extracts		Anti-hyperglycaemic activity	[200]
*Caulerpa mexicana*	Methanolic extracts		Anti-inflammatory activity	[201]

## Data Availability

Not applicable.
